# Repetitive Transcranial Magnetic Stimulation as Maintenance Treatment of Depression: The MAINT-R Randomized Clinical Trial

**DOI:** 10.1001/jamanetworkopen.2025.15881

**Published:** 2025-06-16

**Authors:** Yoshihiro Noda, Masataka Wada, Yu Mimura, Keita Taniguchi, Ryosuke Tarumi, Sotaro Moriyama, Naohiro Arai, Sakiko Tsugawa, Kevin E. Thorpe, Zafiris J. Daskalakis, Hiroyuki Uchida, Masaru Mimura, Daniel M. Blumberger, Shinichiro Nakajima

**Affiliations:** 1 Department of Neuropsychiatry, Keio University School of Medicine, Shinjuku-ku, Tokyo, Japan; 2 Department of Psychiatry, International University of Health and Welfare, Mita Hospital, Mita, Minato-ku, Tokyo, Japan; 3 Shinjuku-Yoyogi Mental Lab Clinic, Sendagaya, Shibuya-ku, Tokyo, Japan; 4 Department of Psychiatry and Behavioral Sciences, Stanford University, Stanford, California; 5 Department of Neuropsychiatry, Faculty of Life Sciences, Kumamoto University, Kurokami Chuo-ku, Kumamoto, Japan; 6 Dalla Lana School of Public Health, University of Toronto, Toronto, Ontario, Canada; 7 The Hospital for Sick Children, Toronto, Ontario, Canada; 8 Department of Psychiatry, University of California, San Diego Health, La Jolla; 9 Temerty Centre for Therapeutic Brain Intervention, Campbell Family Research Institute, Centre for Addiction and Mental Health, Toronto, Ontario, Canada; 10 Department of Psychiatry, Temerty Faculty of Medicine, University of Toronto, Toronto, Ontario, Canada

## Abstract

**Importance:**

Depression relapse poses significant medical and economic challenges. Repetitive transcranial magnetic stimulation (rTMS) as maintenance treatment may prevent relapse of treatment-resistant depression (TRD).

**Objective:**

To compare the effectiveness between low-frequency rTMS and lithium in preventing TRD relapse.

**Design, Setting, and Participants:**

This randomized clinical trial was conducted from September 1, 2018, to May 31, 2023, at Keio University Hospital and Shinjuku-Yoyogi Mental Lab Clinic, Tokyo, Japan, among 75 participants with TRD aged 18 years or older with moderate-to-severe depressive symptoms despite at least 2 adequate antidepressant treatments who subsequently responded to an acute course of bilateral rTMS.

**Interventions:**

Participants were randomly assigned at a 1:1 ratio to receive right dorsolateral prefrontal 1-Hz rTMS (24 weekly sessions; 120% of the resting motor threshold, 900 pulses in 15 minutes) or 24-week maintenance treatment with lithium pharmacotherapy. Participants were maintained on the same venlafaxine dose (150-225 mg/d) as the acute-phase dose.

**Main Outcomes and Measures:**

The primary outcome was the between-group difference in baseline-adjusted Montgomery-Åsberg Depression Rating Scale (MADRS) scores (range, 0-60, where 0 indicates no symptoms and 60 indicates most severe symptoms) at week 24, which was analyzed using a linear mixed-effects model for repeated measures in an intention-to-treat sample. The secondary outcome was the time to relapse (defined as a MADRS score ≥22), which was analyzed using Kaplan-Meier survival curves. Adverse events were also compared between groups.

**Results:**

Among the 75 participants, 38 were assigned to the rTMS group (mean [SD] age, 44.1 [11.7] years; 21 male participants [55.3%]; baseline mean [SD] MADRS score, 8.9 [4.7]), and 37 were assigned to the lithium group (mean [SD] age, 44.1 [11.1] years; 19 male participants [51.4%]; baseline mean [SD] MADRS score, 7.9 [4.5]). There was no significant between-group difference in the primary outcome at week 24 (0.3 points [95% CI, −2.7 to 3.3 points]; *P* = .84). Survival analysis showed no meaningful between-group difference in relapse rates. During the 24-week maintenance phase, there were 7 patients who relapsed in each group. There was a higher number of adverse events among participants in the lithium group (n = 16) than in the rTMS group (n = 3; odds ratio, 7.10 [95% CI, 1.84-27.49]; *P* = .005).

**Conclusions and Relevance:**

In this randomized clinical trial, low-frequency rTMS of the right prefrontal cortex as maintenance treatment showed comparable efficacy, as well as better safety and tolerance, compared with lithium. Maintenance low-frequency rTMS could be a promising relapse prevention strategy for patients with TRD.

**Trial Registration:**

Japan Registry of Clinical Trials: jRCTs032180188

## Key Points

**Question:**

How effective is repetitive transcranial magnetic stimulation (rTMS) compared with lithium in preventing relapse of treatment-resistant depression (TRD)?

**Findings:**

This randomized clinical trial of 75 participants with TRD showed that rTMS and lithium had comparable efficacy in preventing TRD relapse, with no significant difference in relapse rates or primary outcome scores. However, the lithium group experienced a higher number of adverse events than the rTMS group (16 vs 3).

**Meaning:**

Low-frequency rTMS as a maintenance treatment demonstrates similar efficacy to lithium; however, it has better safety and tolerance, indicating it as a promising strategy for preventing relapse among patients with TRD.

## Teaser

This randomized clinical trial compares the effectiveness between repetitive transcranial magnetic stimulation and lithium in preventing relapse of treatment-resistant depression.

## Introduction

Relapse of major depressive disorder poses significant medical and economic challenges. Medically, depression relapse increases disability and decreases quality of life,^1^ which may lead to chronic and ultimately treatment-resistant depression (TRD). Economically, TRD is associated with increased direct and indirect costs across all age groups^2^; furthermore, it is positively correlated with financial stress, especially in less affluent demographic groups.^1,3^ Accordingly, effective treatment and relapse prevention strategies for depression are warranted.^4^

Relapse prevention strategies for TRD after an acute treatment response include maintenance treatment with cognitive behavioral therapy,^5^ ketamine,^6^ combination therapy of antidepressants with different mechanisms of action,^7^ augmentation with lithium, other mood stabilizers,^8^ atypical antipsychotics,^9,10^ and continuation or maintenance electroconvulsive therapy (ECT).^11,12^ However, pharmacotherapy may have systemic adverse effects. In addition, ketamine requires strict medication management and has risks of tachyphylaxis and addiction; lithium has a narrow therapeutic safety window and requires regular management of blood levels; atypical antipsychotics involve a risk of abnormal glucose metabolism and tardive dyskinesia with long-term use; and long-term continuation of maintenance ECT may delay recovery of cognitive function. Therefore, alternative maintenance treatment options with fewer adverse effects and easier long-term management are needed.

Repetitive transcranial magnetic stimulation (rTMS) is an established acute treatment of TRD.^13,14^ Novel protocols have been developed to optimize efficacy and capitalize on the minimal invasiveness and favorable safety profile of rTMS.^15,16^ Prevention strategies for relapse after successful rTMS therapy include maintenance pharmacotherapy and rTMS.^17,18^ However, the comparative effectiveness between rTMS and conventional medical treatments in terms of depression relapse prevention has not been conducted.^19,20^ Accordingly, in the Canadian Network for Mood and Anxiety Treatments guidelines, the evidence level for maintenance rTMS as a relapse prevention strategy for TRD is currently level 3.^21^

Three randomized clinical trials (RCTs) have been conducted of maintenance rTMS for relapse prevention.^20,22,23^ All used high-frequency rTMS, with sham stimulation or standard antidepressants as a control. However, to our knowledge, no RCT has examined the effectiveness of maintenance treatment with low-frequency rTMS, which is safer with excellent tolerability. Therefore, we aimed to evaluate the relapse prevention efficacy, tolerability, and adverse events of low-frequency rTMS to the right dorsolateral prefrontal cortex or lithium pharmacotherapy as maintenance treatment for participants with TRD who responded in the Bilateral TMS Effectiveness for Adult Depression (BEAT-D) study.^24,25,26,27^ We hypothesized that rTMS would be as effective as lithium pharmacotherapy. In addition, we preliminarily compared the tolerability and safety of rTMS therapy with that of lithium pharmacotherapy in the maintenance phase by examining the adverse event profile between the 2 treatments.

## Methods

### Trial Design

This was a parallel-group, single-blind (rater-blind) RCT. The study protocol was approved by the Certified Review Board of Keio and registered in the Japan Registry of Clinical Trials (jRCTs032180188) (trial protocol in Supplement 1 and statistical analysis plan in Supplement 2). Written informed consent was obtained from all participants. This study was conducted in accordance with the Declaration of Helsinki.^28^ This RCT was conducted in accordance with the Consolidated Standards of Reporting Trials (CONSORT) reporting guideline. The interval between the acute and maintenance phase was 1 to 2 weeks.

### Participants

This study was conducted from September 1, 2018, to May 31, 2023, at Keio University Hospital and Shinjuku-Yoyogi Mental Lab Clinic, Tokyo, Japan. During the screening interview for this study, the principal investigator or coinvestigators identified the race and ethnicity (Asian, African American, White, and other [Hispanic or Indigenous people]) of each participant based on their observation as part of the clinical epidemiologic information. During the interview at the time of inclusion, the participants’ race and ethnicity were confirmed by observation by the research physicians and by interviewing the participants themselves. The eligibility criterion for the maintenance phase was a Montgomery-Åsberg Depression Rating Scale (MADRS) score (range, 0-60, where 0 indicates no symptoms and 60 indicates most severe symptoms) that improved by 50% or more from baseline to the end of the acute phase (ie, responders). Accordingly, the eligibility criteria were similar to those in the BEAT-D study^29^ as follows: aged 18 years or older; a major depressive disorder diagnosis based on the *Diagnostic and Statistical Manual of Mental Disorders* (Fifth Edition); nonresponsiveness to treatment^30^ (a history of treatment failure with ≥2 previous antidepressants, including 150-225 mg/d of venlafaxine in the lead-in period of the BEAT-D study, defined as a score of ≥3 on the Antidepressant Treatment History Form)^31^; no history of substance use disorders within the previous 6 months; no contraindication to magnetic resonance imaging or TMS; no unstable physical illness or neurologic disorder; no history of seizures or epilepsy; and no cognitive impairment (Mini-Mental State Examination score ≤23).^32^

### Interventions

The rTMS group received 1-Hz rTMS to the right dorsolateral prefrontal cortex (Montreal Neurological Institute coordinates: x = −38, y = 26, z = 44)^33^ (24 weekly 15-minute sessions each involving 900 pulses). The stimulus intensity was set at 120% of the resting motor threshold, with the coil placed in the anterior-posterior direction at a 45° angle to the midline. rTMS was delivered using the MagPro R30 TMS stimulator equipped with a B70 fluid-cooled coil (MagVenture Inc). Among the pharmacotherapy group, lithium was administered as standard treatment until week 24, after extensive adjustment of the dose to achieve blood levels of 0.4 to 0.6 mEq/L (to convert to millimoles per liter, multiply by 1.0)^34,35,36,37^ depending on the patient’s tolerability. Adherence to medication was monitored at 2-week intervals. In both arms, the baseline treatment with 150 to 225 mg/d of venlafaxine continued without dose change.^29^

### Outcomes

The primary outcome measure was the postintervention change in the MADRS score.^38^ The secondary outcomes included the time to relapse in the maintenance phase (MADRS score ≥22)^39^ and the occurrence of adverse events during the 24-week intervention period. In addition, we investigated postintervention changes in the 17-item Hamilton Rating Scale for Depression (HAMD-17) score and 21-item HAMD (HAMD-21)^40^ as well as the Quick Inventory of Depressive Symptomatology, a 16-item self-report Japanese version (QIDS-J).^41^ Cognitive function measures are described in detail in the eMethods in Supplement 3.

### Sample Size Estimation for the Primary Outcome

Given that the minimal clinically meaningful difference in the MADRS score ranges from 1.6 to 1.9 points,^42^ we defined a noninferiority margin wherein the difference in MADRS scores between the rTMS and lithium groups at 24 weeks would be 1.6 points or less. In this context, the rTMS group’s noninferiority to the lithium group would be established if the rTMS group demonstrated no more than approximately 5% inferiority in terms of the effect ratio. Furthermore, as a reference for estimating the sample size in this study, a clinical trial was identified wherein patients with depression who had achieved complete or partial remission after antidepressant treatment were assigned to 1 of 3 treatment groups: (1) combined treatment with rTMS and antidepressants, (2) rTMS monotherapy, or (3) antidepressant monotherapy.^23^ The relapse and recurrence rates across these 3 groups were then compared. The relapse prevention success rate at 24 weeks in the rTMS monotherapy group was approximately 85%, whereas prior literature indicates that the success rate of lithium in preventing relapse and recurrence in depression was approximately 65%.^43^ These figures were subsequently used as the estimated effect ratio for each intervention group. To establish the noninferiority of the rTMS group compared with that of the lithium group in preventing relapse and recurrence (a primary objective of this study), the required sample size was calculated to be 72 participants in total, with 36 participants in each group. This calculation was based on a significance level of .05 and a statistical power of 80% under the assumption that the effect ratio would be 85% and 65% for the rTMS and lithium groups, respectively. The goal was to demonstrate that maintenance rTMS therapy is not inferior to maintenance lithium therapy by a margin of 5% or more in clinical effect. The sample size calculation was conducted using R, version 4.3.0 (R Project for Statistical Computing).

### Randomization, Implementation, and Blinding

Participants were allocated using an adaptive randomization method in a 1:1 ratio to either group using an automated program implemented in R, version 4.3.0, with a custom script by a blinded allocation manager (S.T.). This adaptive approach used weighting for between-group matching of key baseline characteristics, including the number of participants, mean age, variance of age, mean MADRS score, variance of MADRS score, and number of female participants. Information regarding the allocated group was conveyed to the treatment personnel prior to the start of maintenance treatment. All other involved parties, including outcome raters, were blinded to the treatment allocation throughout the study.

### Statistical Analysis

All outcomes were analyzed on an intention-to-treat (ITT) basis. A linear mixed-effects model for repeated measures was used to evaluate between-group differences in the baseline-adjusted estimated marginal mean values of MADRS scores at the 24-week point with a corresponding 95% CI. Because we conducted only a single test for the primary outcome, we did not perform any specific correction for multiple comparisons. Furthermore, we additionally performed post hoc survival analysis to evaluate the time to relapse. The Kaplan-Meier method was used to estimate survival functions, with between-group comparisons using the log-rank test.

In this study, tolerability and safety were evaluated based on the occurrence of self-reported adverse effects during the 24-week intervention period. The incidence of adverse effects was analyzed using the Pearson χ^2^ test and the Fisher exact test for cases in which the expected frequencies were 5 or more and less than 5, respectively. All *P* values were from 2-sided tests, and results were deemed statistically significant at *P* < .05. All statistical analyses were conducted using R, version 4.3.0, with a custom script. The eMethods in Supplement 3 shows the statistical analysis of between-group differences in cognitive function changes during both maintenance treatments.

## Results

### Main Outcomes

**Figure 1.**
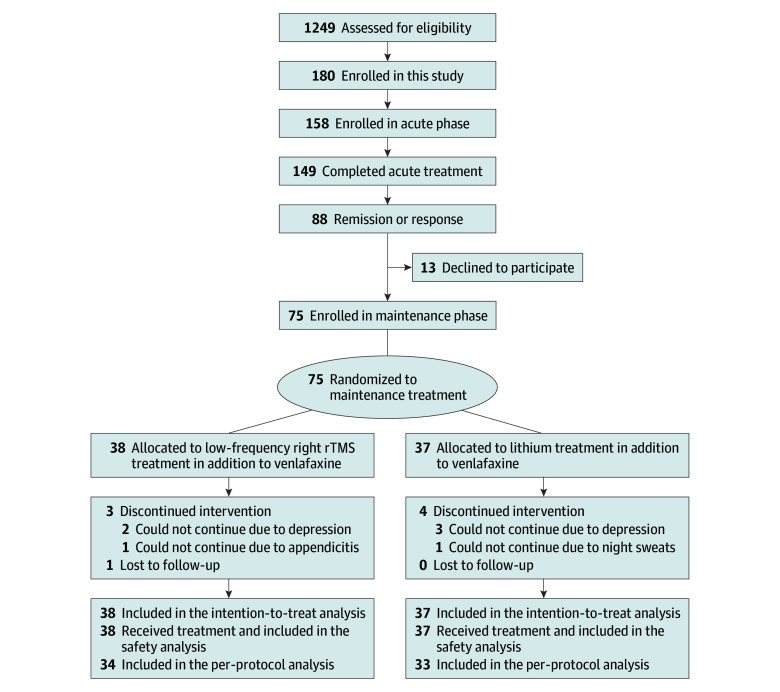
Flowchart for the Maintenance Phase In this study, 88 participants showed remission or response to the acute Bilateral TMS [Transcranial Magnetic Stimulation] Effectiveness for Adult Depression study (BEAT-D study^29^), and 75 of them participated in the maintenance phase of the study. Subsequently, 38 participants were assigned to the low-frequency right dorsolateral prefrontal cortex repetitive transcranial magnetic stimulation (rTMS) treatment group and 37 participants were assigned to the lithium treatment group using adaptive dynamic allocation. The number of participants was not exactly assigned at a 1:1 ratio because the adaptive dynamic allocation method used an algorithm for between-group matching according to age, sex, and depression severity. In the rTMS group, 1 participant was lost to follow-up during the course of the study, and 3 patients discontinued due to adverse events. Therefore, data from 38 participants were included in the intention-to-treat analysis, and data from 34 participants were included in the per-protocol analysis. In the lithium group, 4 participants discontinued due to adverse events during the course of the study. Therefore, data from 37 participants were included in the intention-to-treat analysis, and data from 33 participants were included in the per-protocol analysis.

A participant flowchart is depicted in Figure 1.^29^ Among the 75 participants included in the study, 38 were allocated to the rTMS group (mean [SD] age, 44.1 [11.7] years; 17 female participants [44.7%] and 21 male participants [55.3%]; baseline mean [SD] MADRS score, 8.9 [4.7]; 0 African American participants; 38 Asian participants [100%]; 0 White participants; and 0 participants of other race and ethnicity), and 37 were allocated to the lithium group (mean [SD] age, 44.1 [11.1] years; 18 female participants [48.6%] and 19 male participants [51.4%]; baseline mean [SD] MADRS score, 7.9 [4.5]; 0 African American participants; 36 Asian participants [97.3%]; 1 White participant [2.7%]; and 0 participants of other race and ethnicity); they were all included in the efficacy and safety evaluation (Table 1). A total of 3 of the 37 participants in the lithium group (8.1%) could take only the minimum lithium dose due to tolerance issues.

**Table 1.** Baseline Demographic and Clinical Characteristics

Characteristic	Maintenance 1-Hz rTMS group (n = 38)	Maintenance lithium group (n = 37)
Age, mean (SD), y	44.1 (11.7)	44.1 (11.1)
Age of onset, mean (SD), y	35.4 (12.7)	32.0 (10.9)
Duration of illness, mean (SD), y	8.7 (7.2)	11.8 (8.0)
Duration of treatment, mean (SD), y	7.6 (7.2)	9.9 (6.5)
Duration of education, mean (SD), y	15.5 (2.1)	15.6 (2.3)
Baseline MADRS score, mean (SD)	8.9 (4.7)	7.9 (4.5)
Venlafaxine dose, mean (SD), mg	221.1 (17.0)	212.8 (28.0)
Sex, No. (%)		
Female	17 (44.7)	18 (48.6)
Male	21 (55.3)	19 (51.4)
Prefer not to say	0	0
Race and ethnicity, No. (%)		
African American	0	0
Asian (Japanese)	38 (100)	36 (97.3)
White (US)	0	1 (2.7)
Other (Hispanic or Indigenous people)	0	0
Right-handedness, No. (%)	38 (100)	36 (97.3)
Type of acute treatment, No. (%)		
BL-rTMS	22 (57.9)	22 (59.5)
BL-TBS	16 (42.1)	15 (40.5)

Abbreviations: BL-TBS, bilateral theta-burst stimulation; BL-rTMS, bilateral repetitive transcranial magnetic stimulation; MADRS, Montgomery-Åsberg Depression Rating Scale; rTMS, repetitive transcranial magnetic stimulation.

SI conversion factor: To convert lithium to millimoles per liter, multiply by 1.0.

**Figure 2.**
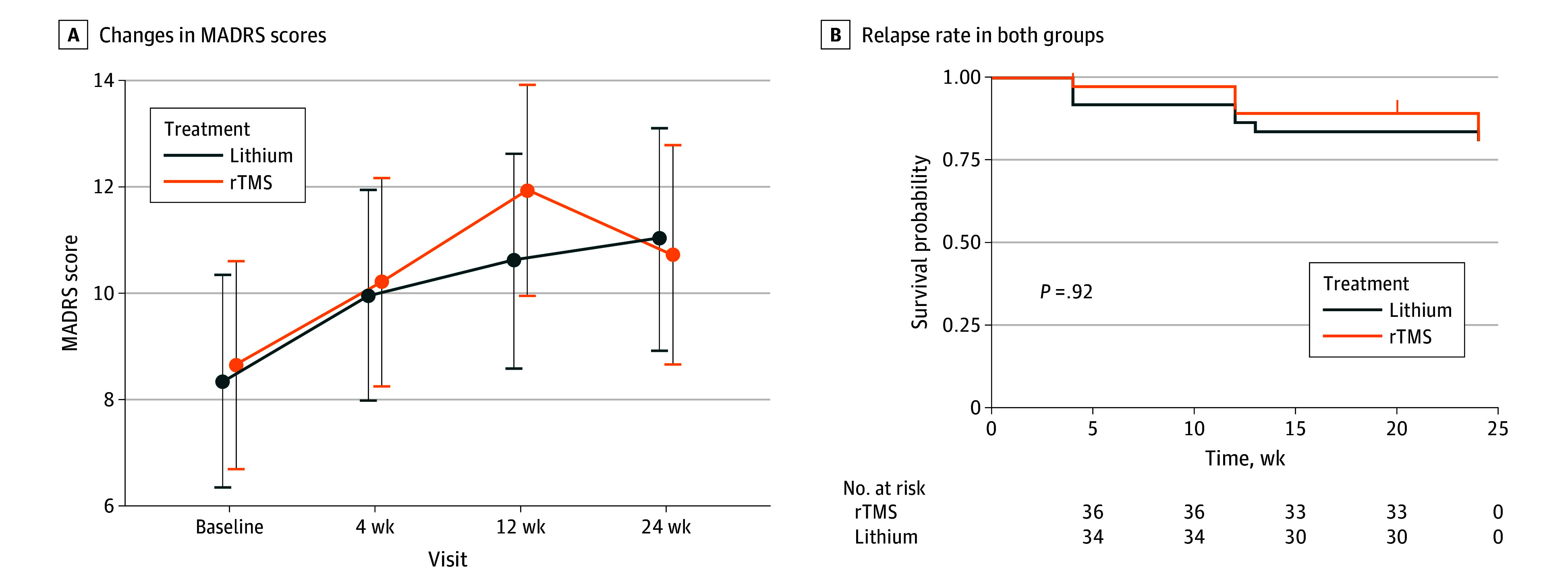
Changes in Depression Scale Scores and Relapse Rates A, Longitudinal changes in Montgomery-Åsberg Depression Rating Scale (MADRS) scores between the repetitive transcranial magnetic stimulation (rTMS) and lithium groups during the maintenance phase: no significant between-group difference was observed in the baseline-adjusted MADRS scores at 24 weeks (0.3 points [95% CI, −2.7 to 3.3 points]; *P* = .84). In addition, there were 7 relapse cases in each group. B, The Kaplan-Meier survival curve evaluating the relapse rate in the rTMS and lithium groups: the Kaplan-Meier estimates did not show any substantial between-group difference in the relapse-free periods. The log-rank test showed no meaningful between-group difference in the survival curves (*P* = .92).

There was no significant between-group difference in the baseline-adjusted MADRS scores at 24 weeks (difference, 0.3 points [95% CI, −2.7 to 3.3 points]; *P* = .84) (Figure 2A and Table 2). There were 7 relapse cases in each group. Furthermore, the log-rank test showed no significant between-group difference in the survival curves (*P* = .92). The Kaplan-Meier estimates also did not indicate any substantial between-group difference in the relapse-free periods (Figure 2B). For the results of the secondary outcome analyses, see eResults and eTable in Supplement 3. There was no significant between-group difference in the baseline-adjusted HAMD-17 scores (difference, 0.3 points [95% CI, −1.4 to 1.9 points]; *P* = .76) (eFigure A in Supplement 3), HAMD-21 scores (difference, 0.6 points [95% CI, −1.1 to 2.4 points]; *P* = .48) (eFigure B in Supplement 3), and QIDS-J scores (difference, 0.51 points [95% CI, −1.2 to 2.2 points]; *P* = .55) (eFigure C in Supplement 3).

**Table 2.** Changes in MADRS Scores During the Maintenance Phase

Time point and arm	Estimated marginal score, mean (SE) [95% CI]
At baseline	
Maintenance rTMS	8.6 (1.0) [6.7-10.6]
Lithium	8.3 (1.0) [6.3-10.3]
At 4 wk	
Maintenance rTMS	10.2 (1.0) [8.2-12.2]
Lithium	10.0 (1.0) [8.0-12.0]
At 12 wk	
Maintenance rTMS	11.9 (1.0) [9.9-13.9]
Lithium	10.6 (1.0) [8.6-12.6]
At 24 wk	
Maintenance rTMS	10.7 (1.1) [8.6-12.8]
Lithium	11.2 (1.1) [8.9-13.1]

Abbreviations: MADRS, Montgomery-Åsberg Depression Rating Scale; rTMS, repetitive transcranial magnetic stimulation.

### Adverse Effects

During the maintenance phase, 3 participants in the rTMS group and 4 participants in the lithium group dropped out of the study before the 24-week assessment for reasons not directly attributable to the treatment. In the rTMS group, 2 participants presented with possible depression relapse and 1 participant presented with appendicitis. In the lithium group, 3 participants presented with possible depression relapse and 1 participant presented with night sweats. There was no significant between-group difference in the dropout rates. Regarding serious adverse events, 1 participant was hospitalized due to worsening depressive symptoms in the lithium group. Adverse events with more than 3 cases were depression (2 cases in the rTMS group and 3 cases in the lithium group), tremor (3 cases in the lithium group), and headache (3 cases in the lithium group). Although there was no evident between-group difference, the number of adverse events was numerically higher in the lithium group than in the rTMS group (16 vs 3; odds ratio, 7.10 [95% CI, 1.84-27.49]; *P* = .005). Specifically, the lithium group had higher incidences of various adverse events, including depression (n = 3), tremor (n = 3), headache (n = 3), dizziness (n = 2), dysesthesia (n = 1), insomnia (n = 1), decreased libido (n = 1), pancreatitis (n = 1), and hypertension (n = 1). The discrepancy between the number of occurrences of adverse events and number of participants can be attributed to cases where a single participant reported multiple adverse events.

## Discussion

Our findings indicated no significant between-group difference in baseline-adjusted MADRS scores at 24 weeks. Moreover, survival analysis indicated no meaningful group difference in relapse, with only approximately 18% of patients in both groups (rTMS, 7 of 38 [18.4%]; lithium, 7 of 37 [18.9%]) experiencing relapse during the 24-week maintenance intervention period. These findings suggest that compared with conventional maintenance pharmacotherapy with lithium, maintenance rTMS treatment may have comparable efficacy as well as better safety and tolerability. Specifically, the incidence of adverse events occurring in 3 or more cases was higher in the lithium group than in the rTMS group.

To our knowledge, this is the first direct head-to-head RCT demonstrating that maintenance rTMS can prevent TRD relapse with efficacy comparable to conventional pharmacotherapy with lithium, which has been demonstrated to be effective maintenance treatment after ECT for TRD.^8^ A unique aspect of our study is that we included patients who were nonresponsive to at least 1 antidepressant treatment other than venlafaxine and standardized them on 150 to 225 mg/d of venlafaxine treatment during the subsequent lead-in period; accordingly, acute bilateral TMS treatment was provided to a relatively homogeneous population with TRD. Another unique characteristic of our study is the use of low-frequency rTMS, which has a safer and more favorable profile than high-frequency rTMS. Accordingly, low-frequency rTMS may be more available in community settings where patients have relatively easy access to treatment. Lithium was selected as the control maintenance treatment because it is well established as a maintenance treatment after ECT. However, long-term lithium therapy requires monitoring of effective blood levels to avoid adverse effects such as tremors, nausea, and diarrhea; such monitoring is not required with rTMS.

A double-blind RCT conducted by Benadhira et al^20^ included 17 participants with TRD who had responded to acute rTMS treatment and compared the relapse prevention effect between the active and sham groups for approximately 11 months. They found that the active group maintained lower HAMD-21 scores than the sham group at 1 and 4 months after the intervention. However, there were no significant between-group differences at the other time points. The previous study may have had limited statistical power given the small number of participants who could start maintenance rTMS. Levkovitz et al^22^ conducted a double-blind RCT of 181 participants with TRD who received deep TMS intervention for a 4-week acute phase and compared the efficacy between the active and sham stimulation groups during the 12-week maintenance phase. They found that response and remission remained stable during the maintenance phase. At the end of the maintenance period, the deep TMS group showed significantly higher response and remission rates than the sham stimulation group. Although this previous study had a sufficient sample size, the duration of the maintenance period was relatively short (12 weeks). Wang et al^23^ conducted a 12-month maintenance RCT for 281 participants with depression in complete or partial remission who received rTMS, antidepressants, or rTMS plus antidepressants for relapse prevention. Compared with the antidepressant group, the rTMS plus antidepressant group and rTMS group had a significantly lower risk of relapse. This finding indicated a significant relapse prevention effect in the rTMS group compared with the antidepressant group.

These 3 RCTs applied a tapering approach of high-frequency rTMS, with 1 study using deep TMS and the others using a clustered TMS protocol. Although tapering with high-frequency rTMS was effective in preventing relapse, the relapse rate ranged from 16% to 60%. In contrast, we used weekly low-frequency rTMS as maintenance treatment, which has fewer adverse events than the established high-frequency rTMS and lithium treatment. Moreover, the relapse rate in our study was approximately 18%, which was lower than previously reported values. In addition, we included a relatively large sample size (n = 75) and long follow-up period (24 weeks). Furthermore, we administered lithium, which has established efficacy and cost-effectiveness, in the control group.

A previous study included 66 patients with TRD who showed a partial response to acute high-frequency rTMS treatment to the left dorsolateral prefrontal cortex and assigned them to receive maintenance rTMS, venlafaxine, or rTMS plus venlafaxine.^44,45^ At 12 months, the overall relapse rate was approximately 60%. This is higher than the relapse rate in our study (18%); however, our study had a shorter follow-up period (approximately 6 months).

### Limitations

Our study has several limitations. First, because this maintenance treatment study was an extension of the acute-phase BEAT-D study, the sample size estimation was inherently exploratory and retrospective, resulting in an unavoidable limitation. Second, in this study, the adaptive randomization method was used to balance age, sex, and MADRS scores between the 2 arms. However, other factors, such as duration of illness and treatment, were not included among the key baseline characteristics, leading to clinical differences between the 2 groups. The lithium treatment group had a disease history approximately 2 years longer than that of the rTMS treatment group. Consequently, it cannot be ruled out that these differences may have influenced treatment resistance and prognosis in both groups, representing a limitation of the study. Third, we did not conduct further follow-up after 24 weeks; therefore, we could not determine the prognosis after this period. Fourth, although most participants in the lithium group could maintain an effective therapeutic dose of lithium (approximately 400-800 mg/d [0.4-0.6 mEq/L]), 8.1% of the participants could take only the minimum lithium dose due to tolerance issues. Fifth, although assessments for somatic and psychiatric adverse events were conducted throughout the maintenance phase, regular blood tests, including thyroid function, were not implemented. Therefore, the count of adverse events in the lithium group might have been underreported. Sixth, this was a single-blind maintenance intervention study after the acute-phase bilateral TMS protocols in an RCT design. Therefore, although the raters who conducted the clinical evaluations were blinded to the allocation, functional unblinding may have occurred because participants may have reported their assigned intervention. Seventh, we did not include a placebo control group. Eighth, regarding TRD, we included a relatively homogenous TRD population who received standardized treatment with venlafaxine. Furthermore, we included only patients who responded to the acute-phase treatment. Accordingly, these strict eligibility criteria may have impeded the generalizability and applicability of our findings to the heterogeneous population of depression in clinical settings. Ninth, this study was conducted in Japan, which may limit the generalizability of our findings to other populations. However, regular outpatient management and interventions are practiced widely in international settings.

## Conclusions

In this randomized clinical trial, low-frequency rTMS over the right dorsolateral prefrontal cortex showed comparative efficacy, as well as better safety and tolerance, compared with lithium. Low-frequency rTMS as maintenance treatment could thus be a viable option for relapse prevention strategy for patients with TRD.

## Notes

Supplement 1.
Study Protocol

Supplement 2.
Statistical Analysis Plan

Supplement 3.
eMethods.

eResults.
**eFigure.** Longitudinal Changes in the Clinical Outcomes Between the Two Arms During the Maintenance Phase **eTable.** Changes in Each Cognitive Measure During the Maintenance Phase
eReferences.

Supplement 4.
Data Sharing Statement
