# Supplementary material for: Repetitive Transcranial Magnetic Stimulation as Maintenance Treatment of Depression: The MAINT-R Randomized Clinical Trial
Source: JAMA Netw Open. 2025 Jun 16;8(6):e2515881. doi: 10.1001/jamanetworkopen.2025.15881 (PMC12171939; doi:10.1001/jamanetworkopen.2025.15881)
Supplement: Supplement 1. — Study Protocol [file jamanetwopen-e2515881-s001.pdf]

Research Protocol

Title: Development of a Novel Transcranial Magnetic Stimulation Therapy for Treatment-Resistant Depression and Identification of Predictive Factors for Treatment Response

Principal Investigator:

- **Name:** Yoshihiro Noda, MD, PhD
- **Institution:** Keio University Hospital
- **Department:** Department of Psychiatry and Neurology
- **Position:** Associate Professor

Version History:

- Version 1.0: August 14, 2017 (Initial version)
- Version 2.0: December 25, 2018 (Revised)
- Version 2.1: January 30, 2019 (Revised)
- Version 2.2: April 7, 2019 (Revised)
- Version 2.3: December 12, 2019 (Revised)
- Version 2.4: April 6, 2020 (Revised)
- Version 2.5: November 3, 2020 (Revised)
- Version 2.6: June 10, 2021 (Revised)
- Version 2.7: August 15, 2022 (Revised)
- Version 2.8: September 20, 2022 (Revised)
- Version 2.9: December 12, 2022 (Revised)
- Version 3.0: February 2, 2023 (Revised)

Table of Contents

1. Summary 3
2. Implementation Structure 4
3. Background Information 7
4. Objectives 9
5. Study Design 10
6. Criteria for Participant Selection, Exclusion, and Discontinuation 14
7. Treatment Protocol 15
8. Efficacy Evaluation 15
9. Safety Evaluation 16
10. Statistical Analysis 18
11. Criteria for Trial Discontinuation 18
12. Handling of Trial Data 18
13. Direct Access to Source Documents 18
14. Monitoring and Auditing 19
15. Ethical Considerations 19
16. Information Disclosure Related to the Study 20
17. Handling and Preservation of Data and Samples 21
18. Financial Burden on Participants and Insurance Measures 22
19. Publication of Clinical Research Information 23
20. Appendix 23

Summary

1.1 Study Design

1.2 Objectives

This physician-led clinical study aims to develop a novel repetitive transcranial magnetic stimulation (rTMS) therapy for the maintenance phase in patients with treatment-resistant depression.

1.3 Participants

Target Population: Patients with treatment-resistant depression **Inclusion Criteria:**

- Individuals aged 18 or older at the time of consent.
- Diagnosed with major depressive disorder according to DSM-5 criteria.
- MADRS score of 18 or higher indicating severity.

Exclusion Criteria:

- Individuals with organic brain diseases (e.g., moderate or severe intracranial organic lesions, neurodegenerative diseases).
- History of seizures or epilepsy, severe or unstable physical illness.
- Contraindications to TMS or MRI, such as metal implants, pacemakers, or claustrophobia.
- Patients who responded to acute-phase TMS treatment.

1.4 Treatment Protocol

Participants who respond to acute-phase rTMS treatment will be divided into two groups: one receiving maintenance pharmacotherapy with venlafaxine plus lithium, and another receiving maintenance pharmacotherapy (venlafaxine) combined with weekly rTMS treatment. The efficacy of relapse prevention in both groups will be compared.

1.5 Target Enrollment and Study Duration

Estimated Target Enrollment: 80 participants for the maintenance phase treatment.

Study Duration: February 14, 2017, to March 31, 2024.

1.6 Contact Information

- **Name:** Yoshihiro Noda, MD, PhD
- **Institution/Department/Position:** Keio University Hospital, Department of Psychiatry and Neurology, Associate Professor
- **Address:** 35 Shinanomachi, Shinjuku-ku, Tokyo 160-0016, Japan
- **Phone:** +81-3-3353-1211 (Ext. 61857)
- **Fax:** +81-3-5379-0187
- **Email:** yoshi-tms@keio.jp

2 Implementation Structure

2.1 Trial Information

- **Research Title:** Development of a Novel Transcranial Magnetic Stimulation Therapy for Treatment-Resistant Depression and Identification of Predictive Factors for Treatment Response
- **Ethics Committee Approval Number:** 20170214
- **Initial Protocol Creation Date:** January 9, 2018 (Version 1.3)
- **Latest Protocol Revision Date:** February 2, 2023 (Version 3.0)
- **Participants:** Patients with treatment-resistant depression
- **Study Design:** Longitudinal interventional study (single-blind randomized trial with simultaneous parallel control group)

2.2 Principal Investigator / Lead Researcher

- **Name:** Yoshihiro Noda, MD, PhD
- **Institution/Department/Position:** Keio University Hospital, Department of Psychiatry and Neurology, Associate Professor
- **Address:** 35 Shinanomachi, Shinjuku-ku, Tokyo 160-8582, Japan
- **Phone:** +81-3-3353-1211 (Ext. 61857)
- **Fax:** +81-3-5379-0187
- **Email:** yoshi-tms@keio.jp

2.3 Co-Lead Researcher

- **Name:** Ryosuke Kitahata, MD, PhD
- **Institution/Department/Position:** Shinjuku-Yoyogi Kokoro no Labo Clinic, Director
- **Address:** 5-27-5 Sendagaya, Shibuya-ku, Tokyo, Room 302, Links Square Shinjuku 3rd Floor
- **Phone:** +81-3-5357-7385

2.4 Data Management Officer

- **Name:** Hiroyuki Takeuchi
- **Institution/Department/Position:** Keio University School of Medicine, Department of Psychiatry and Neurology, Assistant Professor
- **Address:** 35 Shinanomachi, Shinjuku-ku, Tokyo 160-8582, Japan
- **Phone:** +81-3-3353-1211 (Ext. 62454)

2.5 Statistical Analysis Officer

- **Name:** Ryo Takemura, PhD
- **Institution/Department/Position:** Keio University School of Medicine
- **Address:** 35 Shinanomachi, Shinjuku-ku, Tokyo 160-8582, Japan
- **Phone:** +81-3-3353-1211 (Ext. 62454)

2.6 Data Analysis Officer / Collaborative Research Group

2.6.1 Data Analysis Officer

- **Name:** Yoshihiro Noda, MD, PhD
- **Institution/Department/Position:** Keio University School of Medicine, Department of Psychiatry and Neurology, Associate Professor
- **Address:** 35 Shinanomachi, Shinjuku-ku, Tokyo 160-8582, Japan
- **Phone:** +81-3-3353-1211 (Ext. 62454)

2.6.2 Collaborative Research Group

- **Group Name:** Multidisciplinary Translational Research Group
- **Name:** Daniel M. Blumberger, MD, MSc, FRCPC

- **Institution/Department/Position:** Temerty Centre for Therapeutic Brain Intervention, Centre for Addiction and Mental Health, Department of Psychiatry, University of Toronto, Professor
- **Address:** 1001 Queen Street West, Unit 4, Toronto, ON M6J 1H4, Canada
- **Phone:** +1-416-535-8501 (Ext. 33662)

2.7 Allocation Officer

- **Name:** Sakiko Tsugawa
- **Institution/Department/Position:** Keio University School of Medicine, Department of Psychiatry and Neurology
- **Address:** 35 Shinanomachi, Shinjuku-ku, Tokyo 160-8582, Japan
- **Phone:** +81-3-3353-1211 (Ext. 62454)

2.8 Monitoring Officer

- **Name:** Hiroyoshi Takeuchi
- **Institution/Department/Position:** Keio University School of Medicine, Department of Psychiatry and Neurology, Assistant Professor
- **Address:** 35 Shinanomachi, Shinjuku-ku, Tokyo 160-8582, Japan
- **Phone:** +81-3-3353-1211 (Ext. 62454)

2.9 Auditing Officer

- **Company:** CMIC Co., Ltd.
- **Address:** Hamamatsucho Building, 1-1-1 Shibaura, Minato-ku, Tokyo 105-0023, Japan
- **Phone:** +81-3-6779-8111

2.10 Administrative Coordinator

- **Name:** Hiromi Sanoki
- **Institution/Department/Position:** Keio University School of Medicine, Department of Psychiatry and Neurology, Research Assistant
- **Address:** 35 Shinanomachi, Shinjuku-ku, Tokyo 160-8582, Japan
- **Phone:** +81-3-3353-1211 (Ext. 62454)
- **Fax:** +81-3-5379-0187

2.11 Medical Expert

- **Name:** Yoshihiro Noda

2.12 MRI Examination Institution

- **Institution:** MRI Experimental Facility, Center for Evolutionary Cognitive Sciences, Graduate School of Arts and Sciences, The University of Tokyo
- **Contact Person/Position:** Shinsuke Koike, Associate Professor
- **Address:** 3-8-1 Komaba, Meguro-ku, Tokyo, Japan
- **Phone Number:**

3. Background Information

3.1 Current Status of the Target Disease in Japan and Abroad

In Japan, approximately one million people suffer from depression, and this number has been increasing annually. Depression ranks second among all diseases in terms of disability-adjusted life years, with around 30% of cases being treatment-resistant depression (TRD) (Ministry of Health, Labour and Welfare). Depression has a high prevalence among individuals aged 30-50, significantly affecting their ability to study and work, leading to substantial socioeconomic losses and, in the worst cases, resulting in suicide.

3.2 History and Content of Standard Treatments Implemented

Traditional treatments for depression include pharmacotherapy, psychotherapy, and electroconvulsive therapy (ECT). Recently, repetitive transcranial magnetic stimulation (rTMS) has gained attention as a new treatment option for refractory depression.

3.3 Current Standard Treatment, Efficacy, and Outcomes

Although the optimal parameters for rTMS treatment are still under investigation, the most widely used rTMS protocols in clinical and research settings involve excitatory stimulation with high-frequency 10Hz to the left dorsolateral prefrontal cortex (DLPFC) and inhibitory stimulation with low-frequency 1Hz to the right DLPFC, or combined bilateral DLPFC stimulation. A common issue in depression treatment is the frequent recurrence and relapse after achieving remission through acute-phase treatment (Thase,

2006). Traditional strategies for maintaining remission include maintenance pharmacotherapy with venlafaxine and lithium or maintenance ECT. However, maintenance pharmacotherapy often has limited efficacy (Cox et al., 2012; Gili et al., 2015), and while maintenance ECT provides immediate efficacy, it frequently leads to recurrence and relapse (Youssef and McCall, 2014; Brown et al., 2014). The stigma associated with ECT also has significant psychosocial impacts on patients and their families (Holtzheimer et al., 2012). This study aims to verify the efficacy of maintenance rTMS for TRD.

3.5 Information on Medical Devices Used in Clinical Research

The medical device used in this study is the "MagPro System" (manufactured by Inter Reha Co., Ltd.), which is already certified as a "magnetic stimulation device." However, it is not approved or certified as a "transcranial therapeutic magnetic stimulation device."

3.5.1 Experimental Device

- **Generic Name:** Magnetic Stimulation Device (Code 36902000)
- **Brand Name:** MagPro System (R30), Inter Reha Co., Ltd.
- **Approval Number:** 224AIBZX00013000
- **Medical Device Classification:** Advanced Medical Device (Specially Controlled Medical Device)
- **Manufacturer:** Tonica Elektronik A/S
- **Country of Manufacture:** Denmark

3.5.2 Usage Method and Duration

- **Maintenance rTMS Duration:** Weekly maintenance rTMS for a total of 24 weeks.

3.5.3 Target Population for the Study

- The study will recruit participants diagnosed with depression according to DSM-5 criteria.

3.5.4 Benefits and Risks of Administering rTMS

- **Risks and Countermeasures:** rTMS is a non-invasive treatment paradigm and is considered to pose no greater risk than traditional insurance-covered treatments such as pharmacotherapy. However, rTMS may cause transient local pain or discomfort due to muscle contractions in the head and neck area. Rarely, transient hearing loss may occur due to the clicking sound generated by magnetic stimulation, which can be mitigated by using earplugs during the procedure. There is also a very rare risk of inducing seizures, which can be minimized by excluding participants with a history of seizures or epilepsy during recruitment.
- **Benefits for Participants:** Participants will benefit from receiving the latest rTMS therapy and thorough evaluation of their psychiatric symptoms throughout the study. Additionally, no financial burden related to the research will be imposed on the participants.

3.6 Adherence to Regulations

This study will be conducted in accordance with the research protocol and the following requirements:

- Ethical principles based on the Declaration of Helsinki
- Clinical Research Act (Act No. 16 of 2017)
- Ethical Guidelines for Medical and Health Research Involving Human Subjects
- Relevant laws and regulations (e.g., Personal Information Protection Act, Conflict of Interest Management Guidance in Clinical Research)

4. Objectives

The primary objective of this study is to evaluate the preventive effects of a maintenance repetitive transcranial magnetic stimulation (rTMS) protocol on the recurrence and relapse of depression. A prospective intervention trial will be conducted, ensuring that participants in each maintenance phase arm (conventional maintenance pharmacotherapy group vs. maintenance rTMS treatment group) are matched as closely as possible in terms of age, gender, and severity of depressive symptoms. The efficacy of the clinical effect will be assessed using clinical evaluation scales, such as the Montgomery-Åsberg Depression Rating Scale (MADRS), by evaluators who are blinded to the treatment content. To rigorously define treatment-resistant depression (TRD), the initial phase of medication adjustment will standardize the use of

venlafaxine as the antidepressant, with controlled conditions for changing the type and dosage of antidepressants.

5. Study Design

5.1 Evaluation Items

1. **Primary Evaluation Item**
   - Difference from baseline in the Montgomery-Asberg Depression Rating Scale (MADRS).
2. **Secondary Evaluation Items**
   - Difference from baseline in the following measures: ➢ 17-Item Hamilton Rating Scale for Depression (HRSD) ➢ 16-Item Quick Inventory of Depressive Symptoms-Japanese version (QIDS16-J)
3. **Exploratory Evaluation Items**
   - In addition to primary and secondary evaluation items, changes in the following: ➢ Montreal Cognitive Assessment (MoCA) ➢ Repeatable Battery for the Assessment of Neuropsychological Status (RBANS) Japanese version ➢ Stroop Neuropsychological Screening Test (SNST) ➢ Trail Making Test (TMT) ➢ The Executive Interview (EXIT25)
4. **Safety Evaluation Items**
   - Incidence of adverse events and malfunctions from the time of consent to the end of the final observation.

5.2 Study Methods

This study will be conducted on patients with depression who meet the selection criteria. It will adhere to the ethical principles based on the Declaration of Helsinki, protecting the rights and welfare of the patients. Participation in this study is voluntary, and for minors or those without consent capacity, the decision will be made by their legal representatives. Not consenting to participate will not result in any disadvantage, including changes to their treatment.

At Keio University School of Medicine's Department of Psychiatry and Neurology outpatient clinic or in a secure conference room or interview room, the attached explanatory document titled "Development of a Novel Transcranial Magnetic Stimulation Therapy for Treatment-Resistant Depression and Identification of

Predictive Factors for Treatment Response" will be provided to the participant. Based on this document, the purpose of the study, voluntary nature of participation and freedom to withdraw, study methods, participant cooperation details, benefits and disadvantages for the participants, protection of personal information, disclosure of the research protocol, disclosure of results to participants, publication of research outcomes, ownership of intellectual property arising from the research, policy for handling materials after the study ends, cost burden, and contact information will be explained. Consent will be obtained after confirming that the participant fully understands these aspects.

(1) Clinical Evaluation Items and Scales:

- Participant Background: Gender, age, weight, height, duration of illness, smoking history, medical history, medication history, past history.
- Antidepressant Treatment History Form
- Transcranial Magnetic Stimulation Adult Safety Screen
- Diagnosis: DSM-5
- Psychiatric Symptoms: HRSD21, MADRS, QIDS16
- Cognitive Function: MoCA, RBANS, SNST, TMT, EXIT25

Maintenance treatment will include only those who have shown a ≥50% improvement in MADRS scores during acute-phase treatment. Participants will be randomly assigned to:

1. Maintenance pharmacotherapy group with conventional venlafaxine and lithium combination.
2. Maintenance pharmacotherapy (venlafaxine) plus weekly 1Hz-right DLPFC maintenance rTMS group.

Maintenance rTMS treatment will continue for 24 weeks.

5.3 Bias

To minimize biases, the assignment manager will use adaptive randomization based on factors such as age, gender, and severity of depression to allocate participants into the maintenance phase.

5.4 Participant Enrollment and Observation Period

Estimated Target Enrollment: 80 participants for maintenance treatment. The final registration deadline is July 2022 to ensure the observation of the last participant is completed by the study end date of March 31, 2024. **Study Duration:** February 14, 2017, to March 31, 2024.

5.5 Criteria for Partial or Complete Discontinuation of the Study

The study will end if any of the following occur:

1. The study end date of March 31, 2024, is reached.
2. The last participant's tests and evaluations are completed, data is fixed, and a report is prepared.
3. Significant information is obtained regarding the quality, safety, or efficacy of the treatment.
4. Participant recruitment is difficult, making it challenging to achieve the planned number of cases.
5. Instructions for changes to the study protocol are received from the ethics review committee, and it is difficult to accept these changes.
6. The ethics review committee recommends or instructs discontinuation.

In such cases, the principal investigator will consult with the co-investigators to decide whether to continue implementing the research plan. When it is decided to suspend or discontinue the study, the principal investigator will promptly report the reasons in writing to the Dean of the School of Medicine and the ethics review committee.

5.6 Management of Experimental Equipment

The "MagPro System R30" medical device used in this study will be used exclusively for this study and will not be used for other research or routine insured medical care. It will be stored and managed strictly under the supervision of the principal investigator and co-investigators.

Maintenance and inspection will be conducted in accordance with the instructions in the attached document.

5.7 Randomization Procedure

The assignment manager will use adaptive randomization to minimize biases related to age, gender, and MADRS score severity when allocating participants into both groups for the maintenance phase intervention.

5.8 Identification of Source Documents and Raw Data

Digital data obtained from each test, evaluation scales used in evaluation items, medical history, and other basic information will be defined as raw data. Source documents will include medical records, consent forms, and worksheets prepared for this clinical research. Data for aggregation and analysis will be derived from the worksheet data. Any discrepancies or inconsistencies among the source documents will be managed to avoid conflicts, and if they arise, the reasons for the discrepancies and the basis for the data used for aggregation and analysis will be documented on the worksheet with the signature and date of the person recording.

In this study, a copy of the worksheet will be treated as the case report form. During the creation of the worksheet copy (case report form), copies will be made for each completed page, ensuring that the contents are identical to the original. The copy creator's signature and date will be added. If multiple pages are copied at once, they will be consolidated (stapled and bound with bookbinding tape), and the first page will be signed and dated.

Case report forms created at Keio University Hospital and other participating research institutions will be collected by the monitoring personnel during each monitoring session and submitted to the data management supervisor. If the frequency of case report form collection significantly decreases due to participants' visit schedules, the principal investigators at each institution may submit the forms to the data management supervisor through the coordination management staff. A record of transfer between the submitter and the receiver will be created and maintained for these case report form collections.

5.9 Protection of Participants' Privacy

Data used in the research will be assigned a consecutive registration number immediately after collection by the personal information manager, excluding information with personal identification codes. The correspondence table will be strictly managed in a locked locker in the research lab of the Department of Psychiatry and

Neurology at Keio University School of Medicine. All obtained data will be aggregated into various electronic files and stored on computers with set passwords, managed under research numbers, and handled separately from personal information (such as participants' names, dates of birth, patient numbers, addresses, and other identifiers).

6. Criteria for Participant Selection, Exclusion, and Discontinuation

6.1 Selection Criteria

Criteria for Inclusion in Maintenance Treatment:

1. Participants whose MADRS score improved by more than 50% from baseline after acute-phase treatment.
2. Individuals who can continuously visit Keio University Hospital once a week for six months.

6.2 Exclusion Criteria

1. Individuals with organic brain diseases (e.g., moderate or severe intracranial organic lesions, neurodegenerative diseases).
2. History of seizures or epilepsy.
3. Substance-related disorders within the past six months.
4. Severe or unstable physical illness.
5. Received ECT within the past six months.
6. Contraindications to TMS or MRI, such as metal implants, pacemakers, or claustrophobia.
7. Incompatibility of head, neck, or body size with the MRI scanner.
8. Evident hearing impairment.
9. Other cases deemed inappropriate as participants by the principal investigator or co-investigators.

6.3 Discontinuation Criteria

If any of the following conditions make it difficult to continue the study, the research will be discontinued for the individual participant:

1. Occurrence of a serious adverse event newly caused by rTMS treatment.
2. Newly developed claustrophobia during MRI scanning.

3. Withdrawal of consent to participate in the study by the participant.
4. Absence from rTMS sessions for three consecutive days or more.
5. Medical necessity for study discontinuation as determined by the principal investigator.

In cases where the above discontinuation criteria are met, the principal investigator will promptly decide to discontinue the study.

7. Study Treatment

7.1 Content of the Study Treatment

The stimulation site of the dorsolateral prefrontal cortex (DLPFC) for rTMS [MNI-152: x±38 y+44 z+26] will be identified using an MRI-guided navigation system. Maintenance treatment will include only those who show a ≥50% improvement in the Montgomery-Asberg Depression Rating Scale (MADRS) score after acute-phase treatment. These responders will be randomly assigned to one of two arms: (1) Maintenance pharmacotherapy group (venlafaxine + lithium combination therapy), or (2) Maintenance pharmacotherapy (venlafaxine) + weekly maintenance rTMS treatment group. The treatment effects on depressive symptoms in each arm will be compared. Changes in the content or dosage of pharmacotherapy during the maintenance phase will generally not be made. Maintenance rTMS treatment will continue for a total of 24 weeks. Participants who miss three or more consecutive maintenance rTMS sessions will be excluded from the study.

7.2 Concomitant Therapies

For medications and therapies that could potentially impact the study results, their usage and dosage will not be altered from the regimen maintained prior to study participation.

8. Efficacy Evaluation

8.1 Efficacy Evaluation Indicators

1. **Primary Evaluation Item**
   - Difference from baseline in the Montgomery-Asberg Depression Rating Scale (MADRS).
2. **Secondary Evaluation Items**

- Difference from baseline in the following measures: ➢ 17-Item Hamilton Rating Scale for Depression (HRSD21) ➢ 16-Item Quick Inventory of Depressive Symptoms-Japanese version (QIDS16-J)

3. **Exploratory Evaluation Items**
   - Changes in the following: ➢ Montreal Cognitive Assessment (MoCA) ➢ Repeatable Battery for the Assessment of Neuropsychological Status (RBANS) ➢ Stroop Neuropsychological Screening Test (SNST) ➢ Trail Making Test (TMT) ➢ The Executive Interview (EXIT25)

8.2 Methods for Evaluating, Recording, and Analyzing Efficacy Evaluation Indicators

Prior to the initiation of trial, the principal investigator and co-investigators will conduct video-based training to ensure inter-rater reliability of at least 0.6 for the evaluation scales.

9. Safety Evaluation

9.1 Safety Evaluation Indicators

Adverse events occurring from the time of consent until the end of maintenance rTMS treatment will be collected and recorded in the case report form.

9.2 Methods for Evaluating, Recording, and Analyzing Safety Evaluation Indicators

The incidence of adverse events and malfunctions will be evaluated. The number of occurrences and the number of cases of adverse events and malfunctions will be aggregated for each treatment group.

9.3 Adverse Events

The intervention in this study is considered to involve minimal invasion, comparable to traditional pharmacotherapy for depression, and is expected to result in very few adverse events. *Note: In this study, efforts will be made to collect information on all adverse events. Only those adverse events for which a causal relationship with this study cannot be denied will be reported to the Clinical Research Board (CRB) and the*

Pharmaceuticals and Medical Devices Agency (PMDA) in accordance with the Clinical Research Act.

9.3.1 Definition of Adverse Events (AE)

In this study, an adverse event is defined as any unfavorable medical occurrence in a participant to whom treatment has been administered. This includes any unintended sign (including abnormal laboratory findings), symptom, or disease temporally associated with the treatment.

9.3.2 Definition of Serious Adverse Events (SAE)

A serious adverse event in this study is defined as an adverse event that results in 1. death, 2. a life-threatening condition, 3. hospitalization or prolongation of existing hospitalization for treatment, 4. disability, 5. a condition leading to disability, 6. a condition considered severe in accordance with the above criteria, or 7. congenital disease or abnormality in subsequent generations. Here, "condition" refers to adverse events for which a causal relationship with participation in this study cannot be reasonably denied.

9.3.3 Procedures for Collecting, Recording, and Reporting AEs/SAEs

Response to Adverse Events: In the event of an adverse event, the situation, and the subsequent response will be thoroughly explained to the participant to alleviate anxiety and ensure that appropriate medical measures are promptly taken.

Recording and Reporting: If an adverse event occurs, the principal investigator will evaluate its severity based on the above criteria. Should a serious adverse event occur during the study period, appropriate medical measures will be taken, the intervention for the participant will be discontinued, and the principal investigator will promptly report the event to the head of the research institution. The head of the institution will seek the opinion of the Clinical Research Review Board regarding the adverse event and take necessary actions.

9.4 Observation Period for Participants Following the Occurrence of AEs/SAEs

Since the interventions in this study involve minimal invasion, it is unlikely that adverse events exceeding those in general insurance-covered medical care will occur. Should

clinical issues arise during the study, consultation with an independent clinician will be conducted.

10. Statistical Analysis

Please refer to the Statistical Analysis Plan Document.

11. Criteria for Study Discontinuation

In addition to the criteria stipulated for partial or complete discontinuation of the clinical study, the study will be terminated if any of the following conditions are met:

1. Interim analysis determines whether the study is effective or ineffective, making further continuation ethically or scientifically unjustifiable.
2. Interim analysis reveals significant data loss, making it impossible to obtain appropriate statistical analysis results.

If the above conditions are met, the principal investigator and the lead investigator will discuss and decide to terminate the study. Upon deciding to terminate the study, the principal investigator will promptly report the reasons in writing to the Dean of the School of Medicine and the Clinical Research Review Board.

12. Handling of Study Data

In the event of missing data in key evaluation items such as primary evaluation indicators, the decision to include or exclude the data will be made in consultation with the statistical analysis supervisor. No special measures will be taken for other missing data. Participants who violate significant parts of the research protocol (e.g., lack of consent, registration outside the contract period) or those who withdraw from the study at an early stage or withdraw their participation midway through the study will be excluded based on the statistical analysis plan.

13. Direct Access to Source Documents

The principal investigator and the medical institutions conducting the research will accept monitoring, review by the Clinical Research Review Board, and inspections by regulatory authorities concerning documents and procedures related to this study. All data and documents related to this study will be made available for direct access.

14. Monitoring and Auditing

The principal investigator will appoint a monitoring officer to conduct monitoring. The monitoring officer will carry out monitoring in accordance with the monitoring procedure manual and report the results to the principal investigator in writing. Audit operations are outsourced to CMIC Co., Ltd., and will be conducted in accordance with the audit procedure manual.

15. Ethical Considerations

15.1 Benefits and Risks to Participants

Direct benefits to participants include receiving the latest rTMS treatment and having their psychiatric symptoms thoroughly evaluated throughout the study. Participants will incur no costs related to the research.

Potential risks to participants include the burden associated with various tests and blood draws, which are similar in frequency and content to those performed in standard medical care. Adverse events, such as transient local pain or discomfort, may occur during rTMS treatment but are expected to be temporary and recover quickly. Although there is a rare risk of seizure induction due to magnetic stimulation, this risk is minimized by excluding participants with a history of seizures or epilepsy as per the exclusion criteria.

15.2 Informed Consent

Prior to data collection, the principal investigator or co-investigators will use an explanatory document and consent form approved by the Clinical Research Review Board of Keio University School of Medicine. The study's purpose and details will be thoroughly explained to the participant or legal representative, both in writing and verbally. Participation is voluntary, and non-consent will not result in any disadvantages, including changes to treatment content.

At the Keio University School of Medicine's Department of Psychiatry and Neurology outpatient clinic or in a secure conference room or interview room, the attached explanatory document will be provided to the participant or legal representative. This document will cover the study's purpose, voluntary nature of participation and freedom

to withdraw, study methods, participant cooperation details, benefits and risks, protection of personal information, disclosure of the research protocol, publication of research outcomes, ownership of intellectual property resulting from the research, handling of materials after the study, cost burden, and contact information. Written consent will be obtained from participants or guardians who fully understand these aspects, with signatures and dates on the consent form. The principal investigator or co-investigators will keep the original consent form and provide a copy to the participant or guardian. If new information arises during the study that may affect the participant's willingness to continue, the principal investigator or co-investigators will promptly inform the participant and document this communication.

Creation of Consent and Explanatory Documents: Refer to the attached explanatory document and consent form.

Timing and Method of Obtaining Consent: Patients visiting or hospitalized in the Department of Psychiatry and Neurology at Keio University Hospital for diagnosis and treatment will be provided with detailed explanations of the study using the explanatory document by the applicants or co-investigators. Written consent will be obtained from the patient or guardian before data collection begins.

The method of obtaining consent is as follows:

1. Provide an explanation of the study.
2. Document the explanation.
3. Obtain the signed consent form from the participant or legal representative.

Revision of Consent or Explanatory Documents: If new serious adverse events become apparent during the study, requiring a re-explanation

16. Information Disclosure Regarding the Research

Upon completion of the special clinical research transition procedures, this study will be promptly registered in the Japan Registry of Clinical Trials (jRCT).

16.1 Handling Inquiries from Participants and Their Associates

Inquiries from participants and other stakeholders will be addressed by the principal investigator at each participating medical institution. For general inquiries about the study, the following contact person will be responsible:

- **Name:** Yoshihiro Noda, MD, PhD
- **Institution/Department/Position:** Keio University School of Medicine, Department of Psychiatry and Neurology, Associate Professor
- **Address:** 35 Shinanomachi, Shinjuku-ku, Tokyo 160-0016, Japan
- **Phone:** +81-3-3353-1211 (Ext. 61857)
- **Fax:** +81-3-5379-0187
- **Email:** yoshi-tms@keio.jp

16.2 Conflict of Interest

This study is supported by research funds from corporations and private foundations. Potential conflicts of interest that must be reported include research funding for investigator-initiated clinical research from Teijin Pharma, and equipment-in-kind support for this study from Magventure Inc. and Inter Reha Co., Ltd.

17. Handling and Storage of Data and Samples

17.1 Handling and Storage of Data and Samples

The principal investigator is responsible for the strict management of samples and documents related to the research (e.g., copies of various application forms and reports, participant identification code lists, consent forms, case report forms, and other essential documents or records ensuring data reliability). These materials will be stored for at least 5 years from the date of the final research report or 3 years from the final research result report, whichever is later, or according to the regulations of the implementing medical institution.

The correspondence table linking personal information and research IDs will be strictly managed by the personal information protection officer and will be discarded 5 years after the initial publication of the research results. An anonymized final version of the electronic database, with personal information removed, will be strictly managed and stored by the principal investigator for statistical analysis. Even if participants consent to data disclosure, consent may be withdrawn up to 5 years from the final research

report date or 3 years from the final research result report date, whichever is later. Beyond this point, the correspondence table between research IDs and personal information will be discarded, making the deletion of publicly available data impossible.

17.2 Potential Use of Data and Samples in Future Research and Provision to Other Institutions

All data, including personal information, will be handled in such a way that there is no dissemination. Raw data, excluding information with personal identification codes, will be sent to the data analysis collaborative research group via HDD for analysis. The data analysis collaborative research group's method will be consistent with Keio University's.

The principal investigator or an appointed assistant will create records regarding the provision of data once a year in electronic format, which will be stored under the principal investigator for 5 years from the final date of provision. Some research data will be converted into a format that cannot identify individuals and will be registered in a public database in the future.

Individual survey forms will be strictly stored under the personal information manager until there is no longer a need to retain them, such as when the research results are published. Upon becoming unnecessary, they will be discarded. Anonymized data sets will be strictly stored under the principal investigator for potential secondary analysis. If the data is used for research purposes entirely different from the original purpose of this study, an application for review will be submitted to the Clinical Research Review Board.

18. Financial Burden on Participants and Insurance Measures

Participation in this clinical study does not impose any financial burden on the participants. Additionally, a compensation of 15,000 yen will be provided to each participant for their cooperation in the study. Patients who typically receive standard insurance-covered medical care at Keio University Hospital will not become subject to mixed medical treatment by participating in this study.

In the event of any health damage related to the implementation of this study, and if the participant or their family requests compensation, the medical expenses for treating the health damage will be covered either by the research funds of the Department of Psychiatry and Neurology, Keio University School of Medicine, or by insurance related to clinical research.

19. Publication of Clinical Research Information

The results of this study will be promptly presented at relevant academic conferences and submitted to peer-reviewed English journals for publication as soon as possible after the study concludes.

20. Appendix

This study, which obtained approval from the Ethics Committee of Keio University School of Medicine in February 2017, has been ongoing. With the enactment of the Clinical Research Act in April 2018, the study transitioned to a special clinical research. By collaborating with other institutions, such as Dr. Daniel Blumberger at the Centre for Addiction and Mental Health (CAMH) affiliated with the University of Toronto, the feasibility of the study is enhanced, and it is possible to achieve a sufficiently large sample size.
