# Supplementary material for: Repetitive Transcranial Magnetic Stimulation as Maintenance Treatment of Depression: The MAINT-R Randomized Clinical Trial
Source: JAMA Netw Open. 2025 Jun 16;8(6):e2515881. doi: 10.1001/jamanetworkopen.2025.15881 (PMC12171939; doi:10.1001/jamanetworkopen.2025.15881)
Supplement: Supplement 2. — Statistical Analysis Plan [file jamanetwopen-e2515881-s002.pdf]

Version 1.1 (2023/2/28)

Statistical Analysis Plan

Development of a Novel Transcranial Magnetic Stimulation Therapy for Treatment-Resistant Depression and Identification of Predictive Factors for Treatment Response

Principal Investigator:

Yoshihiro Noda, MD, PhD
Department of Psychiatry and Neurology Keio University Hospital

Statistical Analysis Supervisor:

Ryo Takemura, PhD
Keio University School of Medicine

Revision History:

- Version 1.0: Created on October 20, 2018
- Version 1.1: Created on February 28, 2023

Keio University

Version 1.1 (2023/2/28)

Table of Contents

1. Objectives of the Specific Clinical Study 3
   1.1. Primary Evaluation Items (Maintenance Phase) 3
   1.2. Secondary Evaluation Items (Maintenance Phase) 3
2. General Matters in Statistical Analysis 3
   2.1. Data Monitoring and Transformation 3
   2.2. Handling of Missing Values 4
   2.3. Significance Level and Confidence Level 4
   2.4. Subpopulations 4
3. Analysis Population 4
   3.1. Definition of the Analysis Population 4
   3.1.1. Full Analysis Set (FAS) 4
   3.1.2. Per Protocol Set (PPS) 4
   3.1.3. Safety Analysis Population 5
   3.2. Correspondence with Statistical Analysis Items 5
4. Breakdown of Study Participants and Analysis Plan for General Exposure Status 5
   4.1. Breakdown of Study Participants 5
   4.2. Tabulation of Discontinuations or Interruptions 5
   4.3. Data Sets for Analysis 5
5. Analysis Plan for Participant Background and Baseline Values 6
6. Efficacy Analysis 6
   6.1. Analysis Plan for Primary Evaluation Items 6
   6.2. Analysis Plan for Secondary Evaluation Items 6
7. Safety Analysis 7
   7.1. Analysis Plan for Adverse Events 7
8. Statistical Analysis Implementation Structure and Environment 7
   8.1. Statistical Analysis Supervisor 7
   8.2. Statistical Analysis Personnel 7
   8.3. Analysis Environment 8

1. Objectives of the Specific Clinical Study

1.1 Primary Evaluation Items (Maintenance Phase)

Clinical Evaluation: Difference from baseline in the Montgomery-Asberg Depression Rating Scale (MADRS).

1.2 Secondary Evaluation Items (Maintenance Phase)

Clinical Evaluation: Difference from baseline in the following measures:

- 17-Item Hamilton Rating Scale for Depression (HRSD-17)
- 16-Item Quick Inventory of Depressive Symptoms-Japanese version (QIDS16-J)

Cognitive Function: Cognitive Function Evaluation: Difference from baseline in the following measures:

- Montreal Cognitive Assessment (MoCA)
- Repeatable Battery for the Assessment of Neuropsychological Status (RBANS)
- Stroop Neuropsychological Screening Test (SNST)
- Trail Making Test (TMT)
- The Executive Interview (EXIT25)

2. General Matters in Statistical Analysis

2.1 Data Monitoring and Transformation

Research data will be anonymized by removing personal information such as names, patient ID numbers, and dates of birth, and managed by individual administrators. Data will be stored on password-protected computers. The principal investigator will manage and store essential documents related to the research (e.g., copies of various application forms and reports, consent forms, and other necessary documents ensuring data reliability). An anonymized portion of the final electronic database, excluding information with personal identification codes, will be sent to the statistical analysis personnel for analysis. The data transmission will be handled solely by authorized personnel and appropriately managed under the supervision of the statistical analysis supervisor. The principal investigator will create records regarding

the provision of data once a year in electronic format, stored under the principal investigator for 5 years from the conclusion of the clinical research. The principal investigator may appoint an assistant to help with the creation of these records. When publishing research results, no information identifying individual research participants will be disclosed. Image data that can identify individuals will not be subject to public release.

2.2 Handling of Missing Values

No special processing will be conducted.

2.3 Significance Level and Confidence Level

The analysis of primary evaluation items will be performed at a 5% significance level.

2.4 Subpopulations

Not applicable.

3. Analysis Population

3.1 Definition of the Analysis Population

The analysis population in this study includes all participants registered in the study.

3.1.1 Full Analysis Set (FAS)

The FAS comprises participants registered in the study who underwent maintenance phase rTMS treatment. However, participants who violated significant elements of the research protocol (e.g., lack of consent, registration outside the contract period) or received insufficient rTMS treatment sessions and dropped out early, or who withdrew from the study midway, will be excluded.

3.1.2 Per Protocol Set (PPS)

The PPS includes participants registered in the study who completed the maintenance phase rTMS treatment period. Participants who violated significant elements of the research

protocol (e.g., lack of consent, registration outside the contract period) or dropped out early or withdrew from the study midway will be excluded.

3.1.3 Safety Analysis Population

The safety analysis population for the maintenance phase includes participants registered in the study who underwent at least one session of maintenance phase rTMS treatment. However, participants who violated significant elements of the research protocol (e.g., lack of consent, registration outside the contract period) will be excluded.

3.2 Correspondence with Statistical Analysis Items

For all efficacy evaluations, the primary analysis will be conducted on the full analysis set (FAS), with the per protocol set (PPS) used as a reference. Safety analysis will be performed on the safety analysis population.

4. Breakdown of Study Participants and Analysis Plan for General Exposure Status

4.1 Breakdown of Study Participants

1. Participants assigned to maintenance pharmacotherapy: 40 individuals from the above.
2. Participants assigned to maintenance rTMS: 40 individuals from the above.

4.2 Tabulation of Discontinuations or Interruptions

- Participants who may drop out or interrupt during the maintenance phase treatment: 20 individuals.

4.3 Data Sets for Analysis

- **Primary Evaluation Items (Maintenance Phase):**
  - **Clinical Evaluation:** Difference from baseline in the Montgomery-Asberg Depression Rating Scale (MADRS).
- **Secondary Evaluation Items (Maintenance Phase):**
  - **Clinical Evaluation:** Difference from baseline in the following measures:
    - 17-Item Hamilton Rating Scale for Depression (HRSD-17)

    - 16-Item Quick Inventory of Depressive Symptoms-Japanese version (QIDS16-J)
  - **Cognitive Function Evaluation:** Difference from baseline in the following measures:
    - Montreal Cognitive Assessment (MoCA)
    - Quality of Life Enjoyment and Satisfaction Scale (Q-LES-Q)
    - Repeatable Battery for the Assessment of Neuropsychological Status (RBANS) Japanese version
    - Stroop Neuropsychological Screening Test (SNST)
    - Trail Making Test (TMT)
    - The Executive Interview (EXIT25)

5. Analysis Plan for Participant Background and Baseline Values

For background information of the study population, continuous variables will generally be calculated for mean, standard deviation, median, interquartile range, minimum, and maximum values. Categorical variables will be calculated for frequency and percentage. Details will be defined in the statistical analysis plan before data finalization.

6. Efficacy Analysis

6.1 Analysis Plan for Primary Evaluation Items

To compare the group differences between the conventional maintenance pharmacotherapy group and the novel maintenance rTMS treatment group during the maintenance phase, Mixed-Model Repeated Measures (MMRM) will be conducted for clinical outcomes, similar to acute-phase treatment. The differences in treatment effects and changes in biological indicators between the two groups will be statistically evaluated.

6.2 Analysis Plan for Secondary Evaluation Items

To compare the group differences between the conventional maintenance pharmacotherapy group and the novel maintenance rTMS treatment group during the maintenance phase, Analysis of Variance (ANOVA) will be conducted for clinical outcomes, cognitive function outcomes, and biological indicator outcomes, similar to acute-phase treatment. The

differences in treatment effects and changes in biological indicators between the two groups will be statistically evaluated.

7. Safety Analysis

7.1 Analysis Plan for Adverse Events

For all participants, adverse events will be observed and recorded during each rTMS session and documented in the case report form (CRF). After the study concludes, a frequency distribution of each adverse event will be compiled to determine if there are statistical differences in frequency distributions based on the intervention content, and to compare the treatment tolerability of each intervention.

7.1.1 Occurrence of Adverse Events

The occurrence of adverse events will be detailed in the CRF.

7.1.2 Occurrence of Side Effects

The occurrence of side effects will also be detailed in the CRF.

7.1.3 Incidence Rate of Adverse Events with Indeterminate Causality

Adverse events for which a causal relationship cannot be ruled out will be detailed in the CRF. After the study concludes, the incidence rate of these adverse events will be reported.

8. Statistical Analysis Implementation Structure and Environment

8.1 Statistical Analysis Supervisor

- **Name:** Ryo Takemura
- **Affiliation:** Clinical Research Promotion Center, Keio University Hospital

8.2 Statistical Analysis Personnel

- **Name:** Masataka Wada

8.3 Analysis Environment

- **Software:** IBM SPSS Statistics, version 25 or higher
- **Software:** R, version 3.5.1 or higher
