# Supplementary material for: Repetitive Transcranial Magnetic Stimulation as Maintenance Treatment of Depression: The MAINT-R Randomized Clinical Trial
Source: JAMA Netw Open. 2025 Jun 16;8(6):e2515881. doi: 10.1001/jamanetworkopen.2025.15881 (PMC12171939; doi:10.1001/jamanetworkopen.2025.15881)
Supplement: Supplement 3. — eMethods. eResults. eFigure. Longitudinal Changes in the Clinical Outcomes Between the Two Arms During the Maintenance Phase eTable. Changes in Each Cognitive Measure During the Maintenance Phase eReferences. [file jamanetwopen-e2515881-s003.pdf]

Supplementary Online Content

Noda Y, Wada M, Mimura Y, et al. Repetitive transcranial magnetic stimulation as maintenance treatment of depression: the MAINT-R randomized clinical trial. *JAMA Netw Open.* 2025;8(6):e2515881. doi:10.1001/jamanetworkopen.2025.15881

eMethods.
eResults.
eFigure. Longitudinal Changes in the Clinical Outcomes Between the Two Arms During the Maintenance Phase
eTable. Changes in Each Cognitive Measure During the Maintenance Phase
eReferences.

This supplementary material has been provided by the authors to give readers additional information about their work.

eMethods.

Outcomes

In this study, trained clinical psychologists administered the Repeatable Battery for the Assessment of Neuropsychological Status (RBANS) [1], Executive Interview 25 (EXIT-25) [2,3], Stroop Neuropsychological Screening Test (SNST) [4,5], Trail Making Test (TMT) [6], and Montreal Cognitive Assessment - Japanese version (MoCA-J) [7,8] immediately before the start of maintenance treatment, at 4 weeks, 12 weeks, and 24 weeks after the start of maintenance treatment (up to 4 points in time).

Statistical methods

For statistical analysis of between-group differences in changes in cognitive measures with maintenance treatment, repeated measures analysis of variance (ANOVA) was performed for each cognitive measure, with time of testing (4 points) as a within-subject factor and intervention (maintenance rTMS vs. lithium treatment) as a between-subject factor. For statistical analysis, IBM SPSS Statistics version 29.0 was used.

© 2025 Noda Y et al. *JAMA Network Open.*

eResults.

The changes in each cognitive measure for both treatment groups during the maintenance phase are shown in eTable 1.

© 2025 Noda Y et al. *JAMA Network Open.*

eFigure. Longitudinal Changes in the Clinical Outcomes Between the Two Arms During the Maintenance Phase. No significant between-group differences were observed in the baseline-adjusted HAMD-17 scores (A: 0.253 points; 95% CI -1.37–1.87, p=0.758), HAMD-21 scores (B: 0.641 points; 95% CI -1.12–2.40, p=0.475), and QIDS-J scores (C: 0.508 points; 95% CI -1.15–2.17, p=0.547).

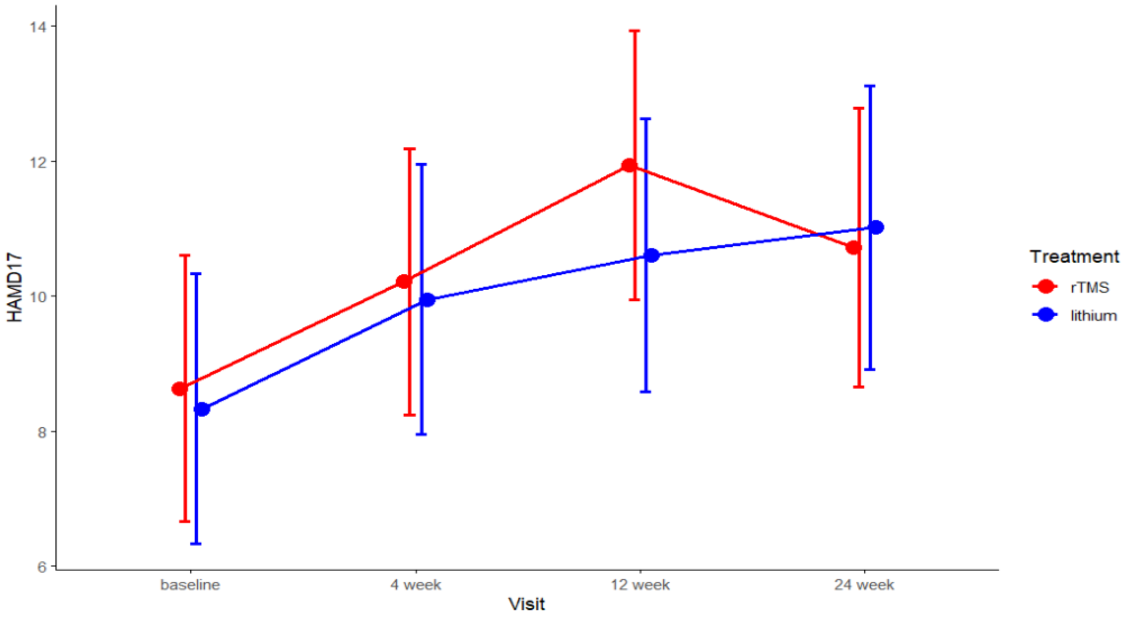

A: HAMD-17

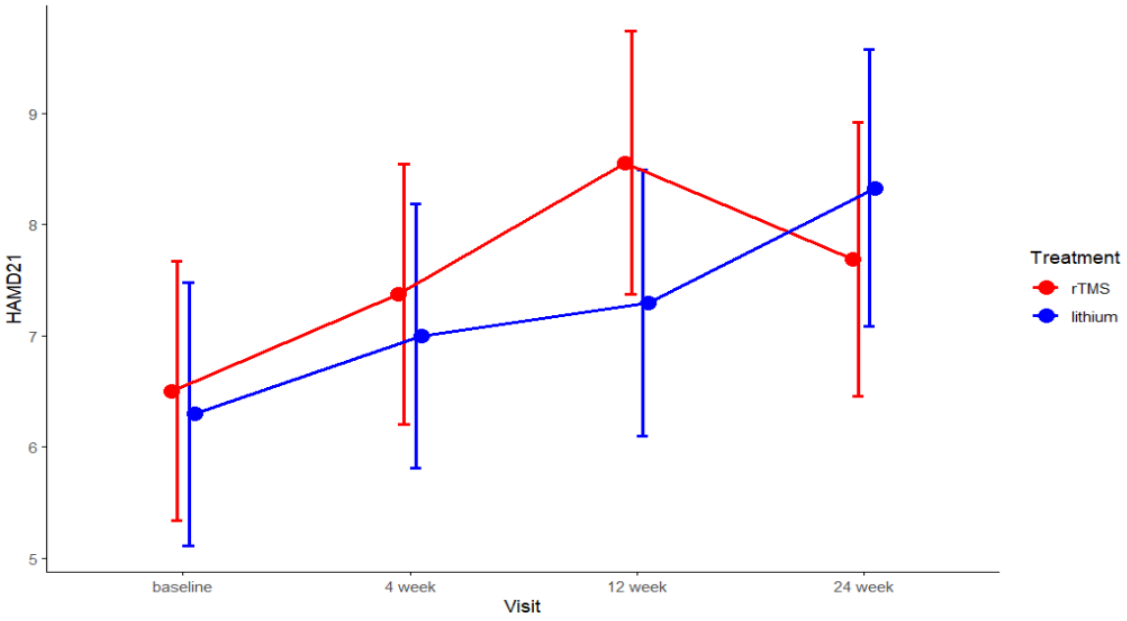

B: HAMD-21

© 2025 Noda Y et al. *JAMA Network Open.*

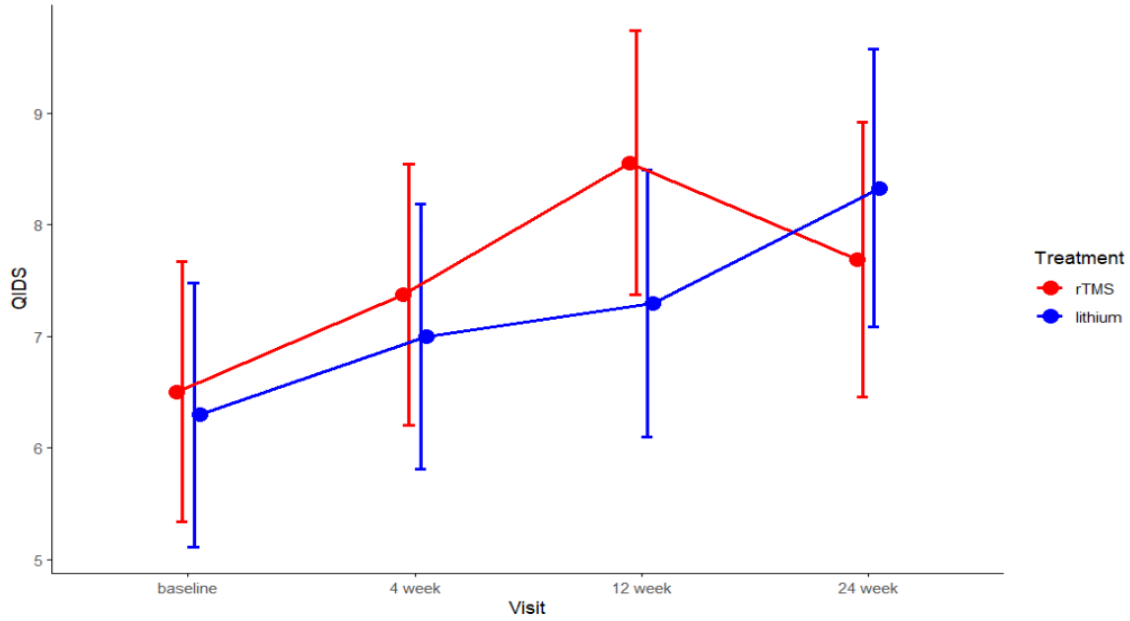

C: QIDS-J

Footnotes: HAMD-17: Hamilton Rating Scale for Depression 17-item; HAMD-21: Hamilton Rating Scale for Depression 21-item; QIDS-J: Quick Inventory of Depressive Symptomatology - a 16-item self-report Japanese version

© 2025 Noda Y et al. *JAMA Network Open.*

eTable. Changes in Each Cognitive Measure During the Maintenance Phase

Time point	Arm	RBANS total scale	EXIT-25 total score	SNST part 3 – part 1	TMT part B – part A	MoCA-J total score
		mean (±SD)				
Baseline	Maintenance rTMS	91.3 (16.6)	3.9 (2.5)	6.1 (4.0)	23.7 (13.9)	26.8 (2.7)
	Lithium	88.4 (16.0)	3.8 (1.6)	5.8 (3.1)	32.3 (33.2)	26.9 (2.6)
4-week	Maintenance rTMS	89.7 (14.4)	3.3 (1.8)	6.1 (3.6)	27.8 (12.0)	27.2 (2.4)
	Lithium	88.7 (13.8)	3.3 (1.4)	6.3 (3.8)	29.6 (16.6)	27.2 (2.4)
12-week	Maintenance rTMS	92.8 (16.1)	3.3 (1.9)	5.4 (4.1)	27.2 (12.0)	27.6 (2.1)
	Lithium	89.9 (13.2)	3.5 (2.2)	6.7 (4.3)	29.4 (13.9)	27.6 (2.4)
24-week	Maintenance rTMS	89.6 (16.5)	3.3 (2.2)	5.7 (3.2)	25.8 (11.6)	27.1 (1.9)
	Lithium	90.5 (17.0)	3.3 (1.7)	5.4 (2.7)	26.9 (15.8)	27.6 (2.2)

Footnotes: RBANS: Repeatable Battery for the Assessment of Neuropsychological Status, EXIT-25: Executive Interview 25, SNST: Stroop Neuropsychological Screening Test, TMT: Trail Making Test, MoCA-J: Montreal Cognitive Assessment - Japanese version, SD: standard deviation

The ANOVA for the RBANS total scale showed no significant main effect of time ($F_{3,195}$=0.991, p=0.398) or time-by-intervention interaction ($F_{3,195}$=1.024, p=0.383). Also, there was no significant main effect of intervention between the groups ($F_{1,65}$=0.319, p=0.574). For the EXIT-25, the ANOVA showed no significant main effect of time ($F_{3,102}$=1.235, p=0.301) or

© 2025 Noda Y et al. *JAMA Network Open.*

time-by-intervention interaction ($F_{3,102}$=0.130, p=0.942), and no significant main effect of intervention between the groups ($F_{1,34}$=0.017, p=0.898). For the SNST (part 3 – part 1), the ANOVA indicated no significant main effect of time ($F_{3,195}$=0.739, p=0.530) or time-by-intervention interaction ($F_{3,195}$=2.007, p=0.114), and no significant main effect of intervention between the groups ($F_{1,65}$=0.392, p=0.534). For the TMT (part B – part A), the ANOVA indicated no significant main effect of time ($F_{3,195}$=0.396, p=0.756) or time-by-intervention interaction ($F_{3,195}$=0.787, p=0.502), and no significant main effect of intervention between the groups ($F_{1,65}$=0.775, p=0.382). For the MoCA-J, the ANOVA showed a significant main effect of time ($F_{3,195}$=3.87, p=0.01) but no significant time-by-intervention interaction ($F_{3,195}$=0.234, p=0.873). Also, no significant main effect of intervention was observed between the groups ($F_{1,65}$=0.219, p=0.642).

© 2025 Noda Y et al. *JAMA Network Open.*

eReferences.

1. Randolph C, Tierney MC, Mohr E, Chase TN. The Repeatable Battery for the Assessment of Neuropsychological Status (RBANS): preliminary clinical validity. *J Clin Exp Neuropsychol*. Jun 1998;20(3):310-9. doi:10.1076/jcen.20.3.310.823

2. Mujic F, Lebovich E, Von Heising M, Clifford D, Prince MJ. The Executive Interview (EXIT25) as a tool for assessing executive functioning in older medical and surgical inpatients referred to a psychiatry service: feasibility of creating a brief version. *Int Psychogeriatr*. Jun 2014;26(6):935-41. doi:10.1017/S104161021400026X

3. Matsuoka T, Kato Y, Taniguchi S, et al. Japanese versions of the executive interview (J-EXIT25) and the executive clock drawing task (J-CLOX) for older people. *Int Psychogeriatr*. Aug 2014;26(8):1387-97. doi:10.1017/S104161021400088X

4. Trenerry MR, Crosson B, DeBoe J, Leber WR, eds. *Stroop neuropsychological screening test manual. Lutz: Psychological Assessment Resources*. Lutz: Psychological Assessment Resources; 1989.

5. Lezak MD, Howieson DB, Bigler ED, Tranel D, eds. *Neuropsychological assessment*. 5th ed. ed. New York: Oxford University Press; 2012.

6. Soukup VM, Ingram F, Grady JJ, Schiess MC. Trail Making Test: issues in normative data selection. *Appl Neuropsychol*. 1998;5(2):65-73. doi:10.1207/s15324826an0502_2

7. Fujiwara Y, Suzuki H, Yasunaga M, et al. Brief screening tool for mild cognitive impairment in older Japanese: validation of the Japanese version of the Montreal Cognitive Assessment. *Geriatr Gerontol Int*. Jul 2010;10(3):225-32. doi:10.1111/j.1447-0594.2010.00585.x

8. Nasreddine ZS, Phillips NA, Bedirian V, et al. The Montreal Cognitive Assessment, MoCA: a brief screening tool for mild cognitive impairment. *J Am Geriatr Soc*. Apr 2005;53(4):695-9. doi:10.1111/j.1532-5415.2005.53221.x

© 2025 Noda Y et al. *JAMA Network Open.*
