# Supplementary material for: Repetitive Transcranial Magnetic Stimulation as Maintenance Treatment of Depression: The MAINT-R Randomized Clinical Trial
Source: JAMA Netw Open. 2025 Jun 16;8(6):e2515881. doi: 10.1001/jamanetworkopen.2025.15881 (PMC12171939; doi:10.1001/jamanetworkopen.2025.15881)
Supplement: Supplement 4. — Data Sharing Statement [file jamanetwopen-e2515881-s004.pdf]

Data Sharing Statement

Noda. Repetitive Transcranial Magnetic Stimulation as Maintenance Treatment of Depression. *JAMA Netw Open*. Published June 16, 2025. doi:10.1001/jamanetworkopen.2025.15881

Data
Additional Information: Trial Registration: Japan Registry of Clinical Trials: jRCTs032180188. https://jrct.niph.go.jp/re/reports/detail/76805
Data available: Yes
Data types: Deidentified participant data
How to access data: The data that support the findings of this study are available from the corresponding author (the principal investigator, YN) upon reasonable request. YN and MW have full access to all the data in the study and take responsibility for the integrity of the data and the accuracy of the data analysis.
When available: With publication

Supporting Documents
Document types: None

Additional Information
Who can access the data: Researchers whose proposed use of the data has been approved.
Types of analyses: A specified purpose.
Mechanisms of data availability: After approval of a proposal with a signed data access agreement.
